# Supplementary material for: Structural basis of RNAPII transcription on the nucleosome containing histone variant H2A.B
Source: EMBO J. 2025 May 30;44(14):4065–87. doi: 10.1038/s44318-025-00473-6 (PMC12264295; doi:10.1038/s44318-025-00473-6)
Supplement: Supplementary file 1 — Appendix [file 44318_2025_473_MOESM1_ESM.pdf]

Appendix View

Structural basis of RNAPII transcription on the nucleosome containing histone variant H2A.B

Munetaka Akatsu[1,2], Rina Hirano[1,2], Tomoya Kujirai[1,3], Mitsuo Ogasawara[1], Haruhiko Ehara[3], Shun-ichi Sekine[3], Yoshimasa Takizawa[1,4], and Hitoshi Kurumizaka[1,2,3]*

[1]Laboratory of Chromatin Structure and Function, Institute for Quantitative Biosciences, The University of Tokyo, 1-1-1 Yayoi, Bunkyo-ku, Tokyo 113-0032, Japan.

[2]Department of Biological Sciences, Graduate School of Science, The University of Tokyo, 1-1-1 Yayoi, Bunkyo-ku, Tokyo 113-0032, Japan.

[3]Laboratory for Transcription Structural Biology, RIKEN Center for Biosystems Dynamics Research, 1-7-22 Suehiro-cho, Tsurumi-ku, Yokohama 230-0045, Japan.

[4]Department of Computational Biology and Medical Sciences, Graduate School of Frontier Sciences, The University of Tokyo, 1-1-1 Yayoi, Bunkyo-ku, Tokyo 113-0032, Japan.

*To whom correspondence should be addressed; E-mail address: kurumizaka@iqb.u-tokyo.ac.jp

The Appendix View includes

	Page number
Appendix Fig. S1	2
Appendix Fig. S2	3
Appendix Fig. S3	4
Appendix Fig. S4	5
Appendix Fig. S5	6
Appendix Fig. S6	7
Appendix Fig. S7	8
Appendix Fig. S8	9

Appendix Fig. S1

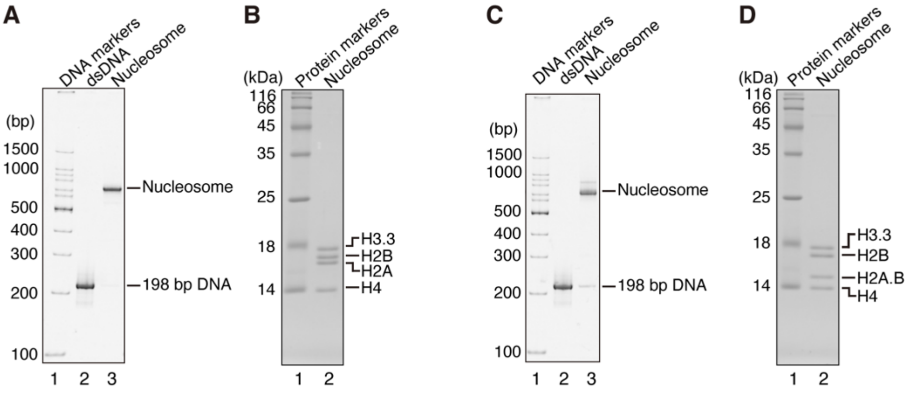

Appendix Fig. S1- The H2A nucleosome and H2A.B nucleosome used in transcription assays and cryo-EM analyses.

(A, B) The H2A nucleosome was analyzed by non-denaturing 6% polyacrylamide gel electrophoresis with ethidium bromide staining (A) and SDS 20% polyacrylamide gel electrophoresis with CBB staining (B). (C, D) The H2A.B nucleosome was analyzed by non-denaturing 6% polyacrylamide gel electrophoresis with ethidium bromide staining (C) and SDS 20% polyacrylamide gel electrophoresis with CBB staining (D).

Appendix Fig. S2

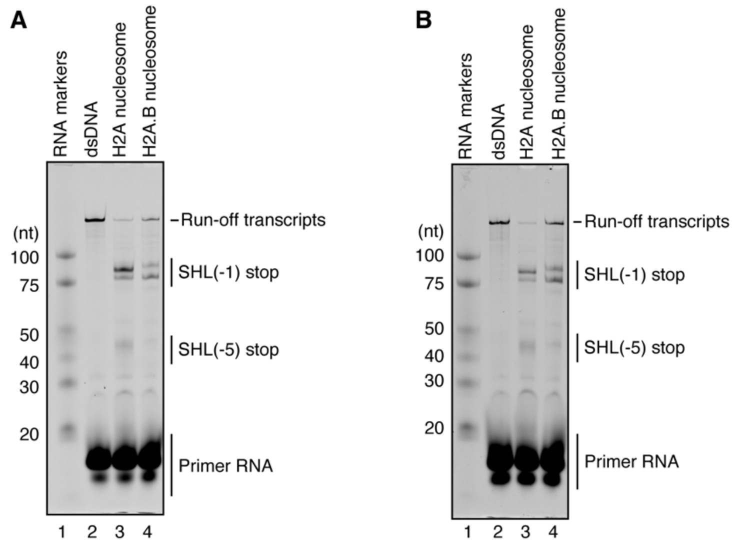

Appendix Fig. S2- Repeated H2A nucleosome and H2A.B nucleosome transcription assays.

(A, B) Replicated experiments of the transcription assay shown in Figure 1C.

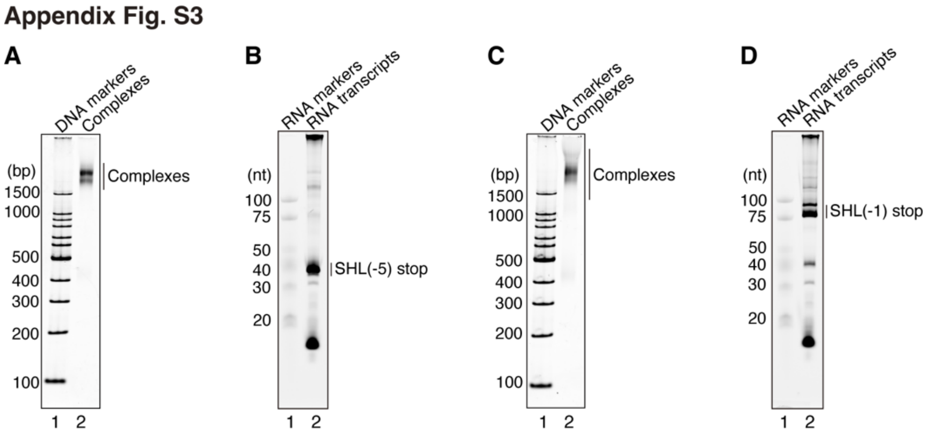

Appendix Fig. S3- The EC-H2A.B nucleosome complexes used in cryo-EM analyses.

(A, B) The EC-H2A.B nucleosome complex paused at the SHL(-5) position for the cryo-EM analysis was analyzed by non-denaturing 4% polyacrylamide gel electrophoresis with SYBR Gold staining (A). RNA transcripts were separated by denaturing 10% polyacrylamide gel electrophoresis and detected with DY647 fluorescent dye (B). (C, D) The EC-H2A.B nucleosome complex paused at the SHL(-1) position for the cryo-EM analysis was analyzed by non-denaturing 4% polyacrylamide gel electrophoresis with SYBR Gold staining (C). RNA transcripts were separated by denaturing 10% polyacrylamide gel electrophoresis and detected with DY647 fluorescent dye (D).

Appendix Fig. S4

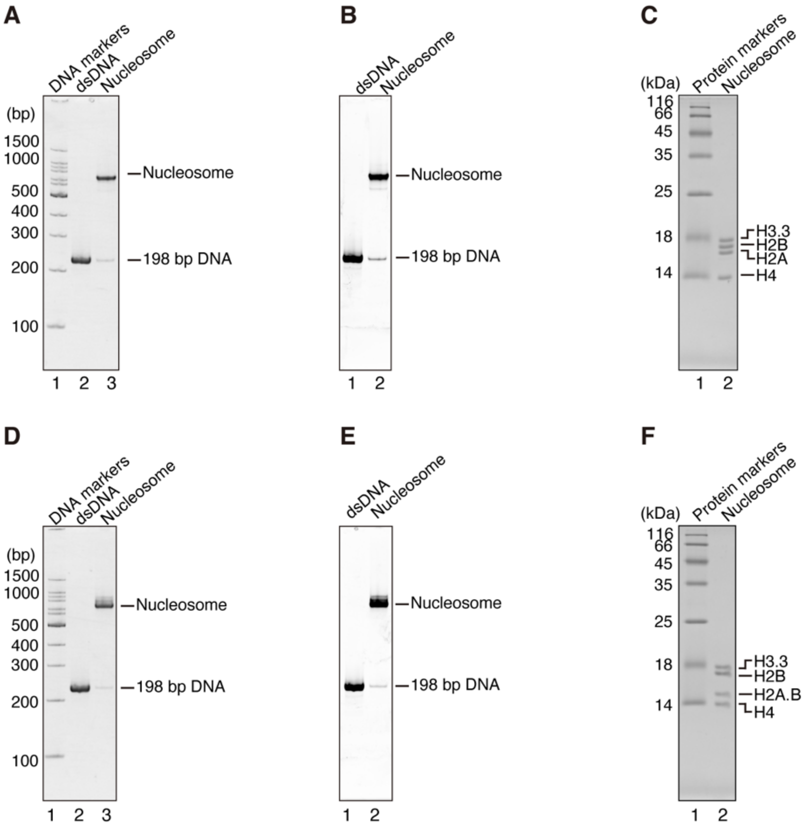

Appendix Fig. S4- The canonical H2A and H2A.B nucleosomes used in DNase I footprinting analysis.

(A-C) The H2A nucleosome was analyzed using non-denaturing 6% polyacrylamide gel electrophoresis with ethidium bromide staining (A) and 6-FAM fluorescent dye detection (B). The histone composition was confirmed by SDS 20% polyacrylamide gel electrophoresis with CBB staining (C). (D-F) The H2A.B nucleosome was analyzed using non-denaturing 6% polyacrylamide gel electrophoresis with ethidium bromide staining (D) and 6-FAM fluorescent dye detection (E). The histone composition was confirmed by SDS 20% polyacrylamide gel electrophoresis with CBB staining (F).

Appendix Fig. S5

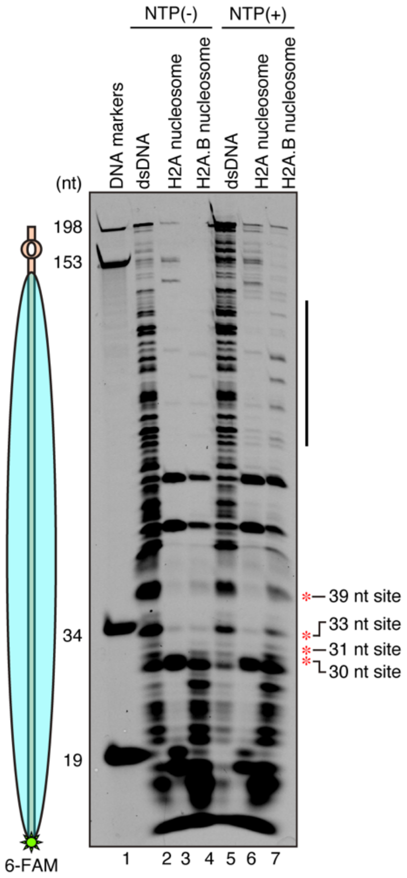

Appendix Fig. S5- Repeated DNase I footprinting analysis.

A replicated experiment of the DNase I footprinting shown in Figure 3B.

Appendix Fig. S6

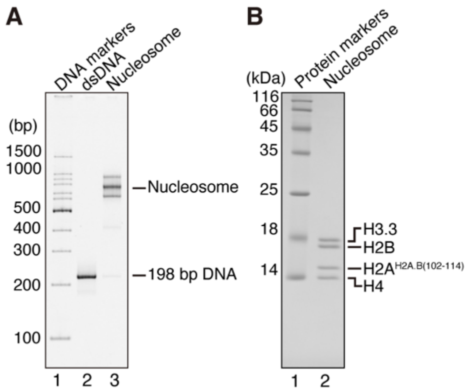

Appendix Fig. S6- The H2A$^{H2A.B(102-114)}$ nucleosome used in transcription assays.

(A, B) The H2A$^{H2A.B(102-114)}$ nucleosome was analyzed by non-denaturing 6% polyacrylamide gel electrophoresis with ethidium bromide staining (A) and SDS 20% polyacrylamide gel electrophoresis with CBB staining (B).

Appendix Fig. S7

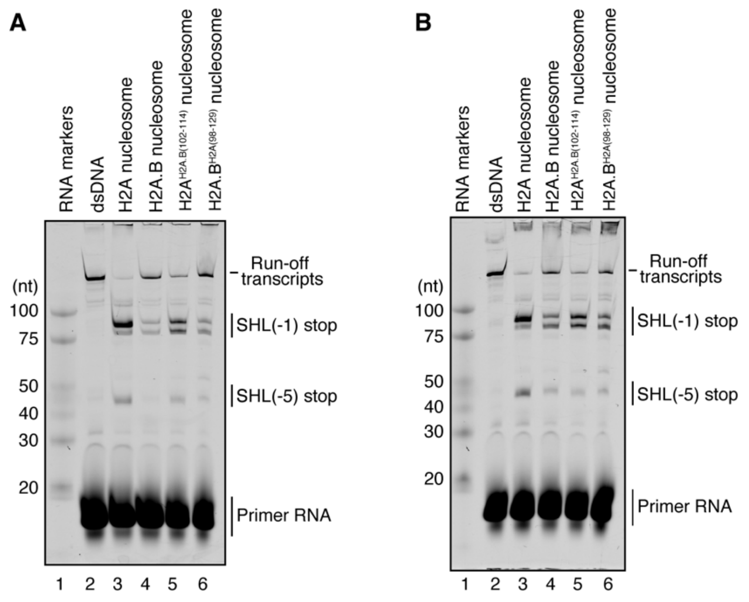

Appendix Fig. S7- Repeated H2A$^{H2A.B(102\text{-}114)}$ nucleosome and H2A.B$^{H2A(98\text{-}129)}$ nucleosome transcription assays.

(A, B) Replicated experiments of the transcription assay shown in Figure 4C.

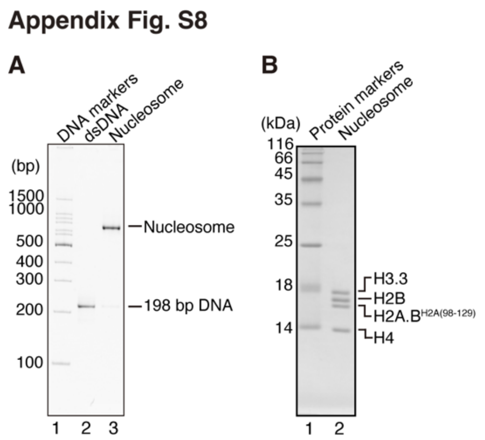

Appendix Fig. S8- The H2A.B$^{H2A.B(98\text{-}129)}$ nucleosome used in transcription assays.

(G, H) The H2A.B$^{H2A(98\text{-}129)}$ nucleosome was analyzed by non-denaturing 6% polyacrylamide gel electrophoresis with ethidium bromide staining (G) and SDS 20% polyacrylamide gel electrophoresis with CBB staining (H).
