# Supplementary material for: Structural basis of RNAPII transcription on the nucleosome containing histone variant H2A.B
Source: EMBO J. 2025 May 30;44(14):4065–87. doi: 10.1038/s44318-025-00473-6 (PMC12264295; doi:10.1038/s44318-025-00473-6)
Supplement: Supplementary file 6 — Expanded View Figures [file 44318_2025_473_MOESM6_ESM.pdf]

Expanded View Figures

Figure EV1. Cryo-EM analysis of the EC-H2A.B nucleosome complex paused at the SHL(−5) position.

(**A**) Representative micrograph of the EC-H2A.B nucleosome complex paused at the SHL(−5) position. Scale bar: 100 nm. (**B**) Representative 2D class averages for the final map of the EC-H2A.B nucleosome complex paused at the SHL(−5) position. Scale bar: 10 nm. (**C**) Workflow of the image processing of the EC-H2A.B nucleosome complex paused at the SHL(−5) position.

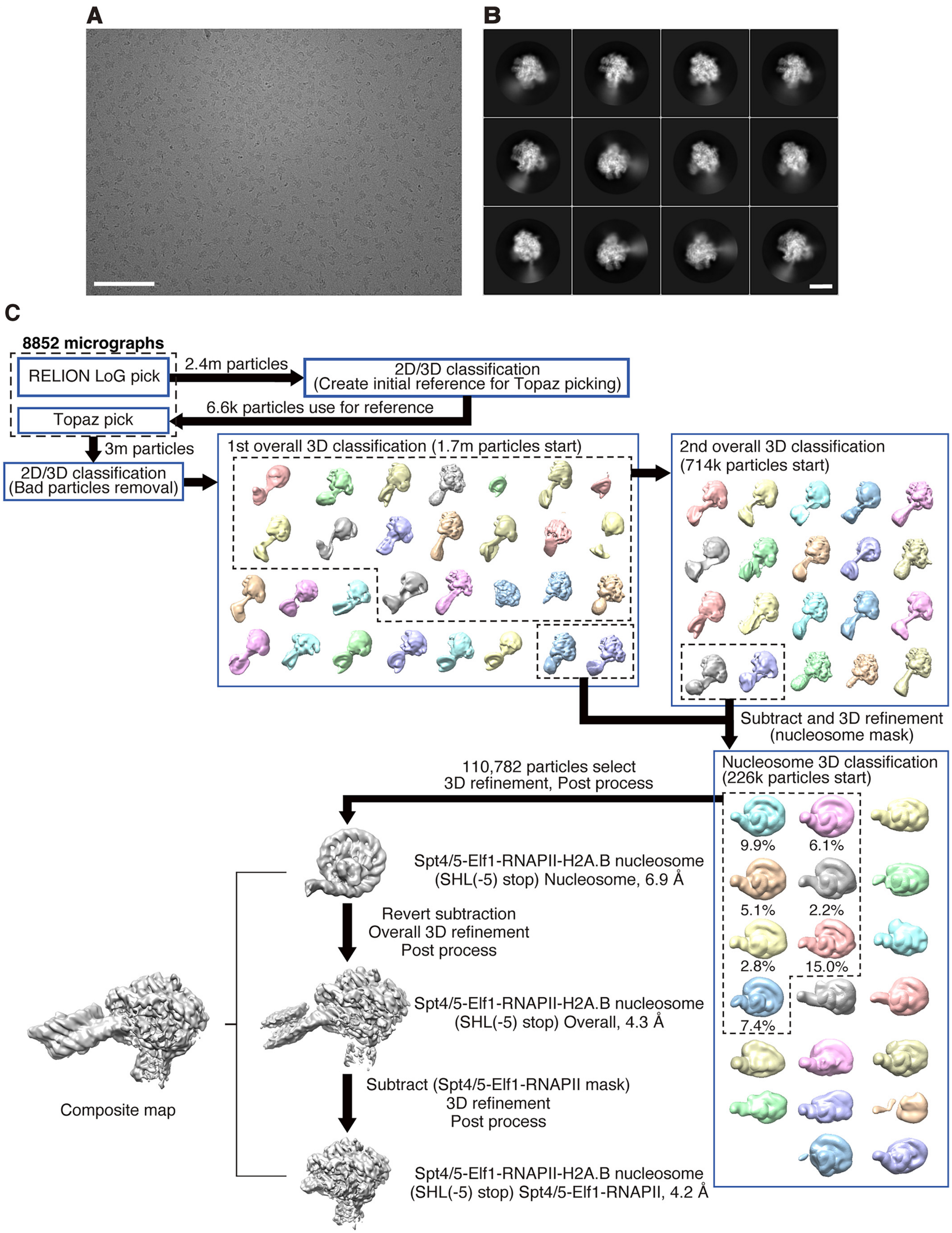

A
B
C
8852 micrographs
RELION LoG pick
2.4m particles
2D/3D classification
(Create initial reference for Topaz picking)
6.6k particles use for reference
Topaz pick
3m particles
2D/3D classification
(Bad particles removal)
1st overall 3D classification (1.7m particles start)
2nd overall 3D classification
(714k particles start)
Subtract and 3D refinement
(nucleosome mask)
Nucleosome 3D classification
(226k particles start)
9.9%
6.1%
5.1%
2.2%
2.8%
15.0%
7.4%
110,782 particles select
3D refinement, Post process
Spt4/5-Elf1-RNAPII-H2A.B nucleosome
(SHL(-5) stop) Nucleosome, 6.9 Å
Revert subtraction
Overall 3D refinement
Post process
Spt4/5-Elf1-RNAPII-H2A.B nucleosome
(SHL(-5) stop) Overall, 4.3 Å
Subtract (Spt4/5-Elf1-RNAPII mask)
3D refinement
Post process
Spt4/5-Elf1-RNAPII-H2A.B nucleosome
(SHL(-5) stop) Spt4/5-Elf1-RNAPII, 4.2 Å
Composite map

 © The Author(s)

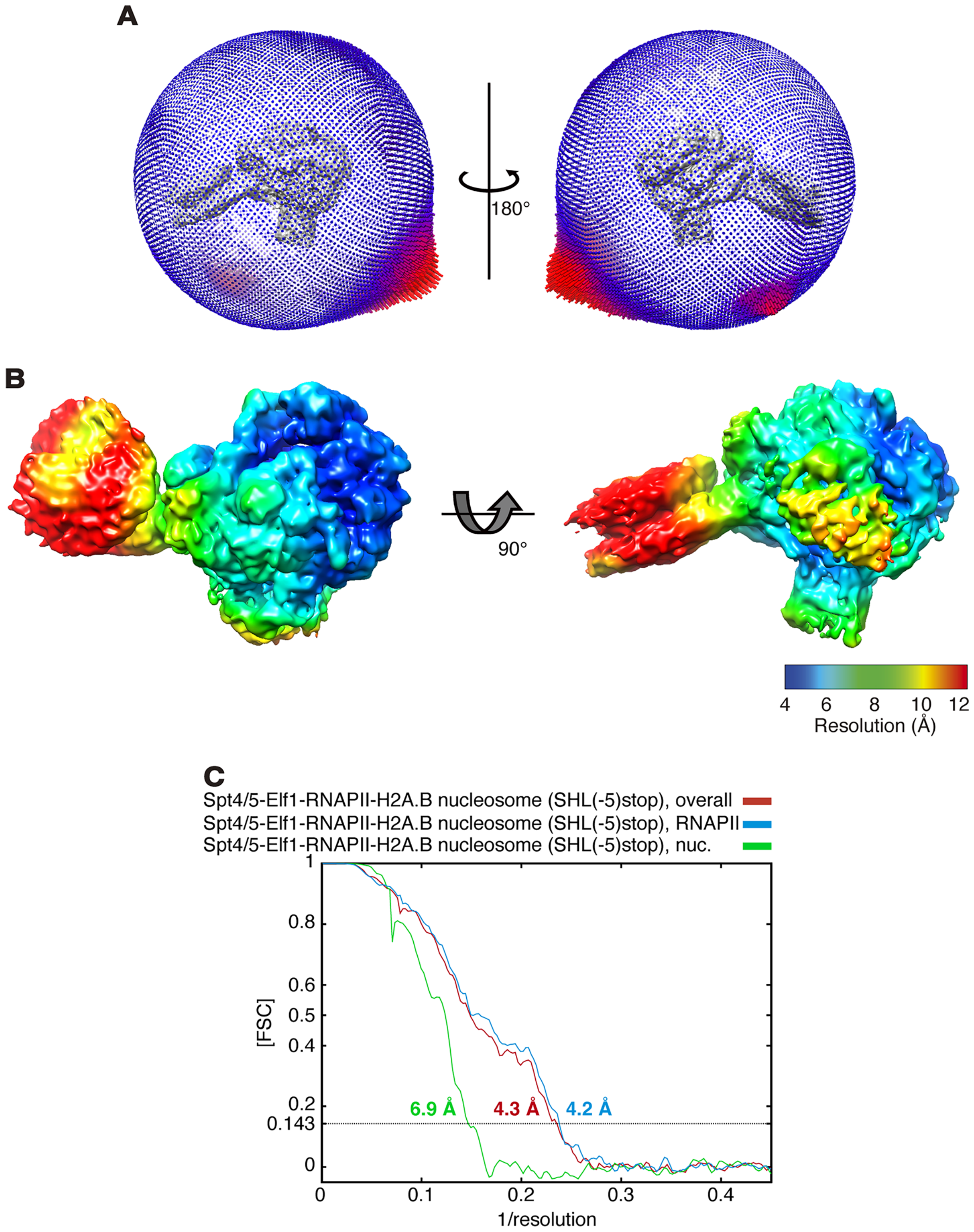

Figure EV2. Cryo-EM map and model qualities of the EC-H2A.B nucleosome complex paused at the SHL(−5) position.

(**A**) Angular distribution of the EC-H2A.B nucleosome complex paused at the SHL(−5) position. (**B**) Local resolution map of the EC-H2A.B nucleosome complex paused at the SHL(−5) position. (**C**) Fourier Shell Correlation (FSC) curve of the EC-H2A.B nucleosome complex paused at the SHL(−5) position. The final resolutions of the structures were estimated at 4.3 Å (overall), 4.2 Å (Spt4/5-Elf1-RNAPII), and 6.9 Å (nucleosome) (FSC = 0.143).

© The Author(s)

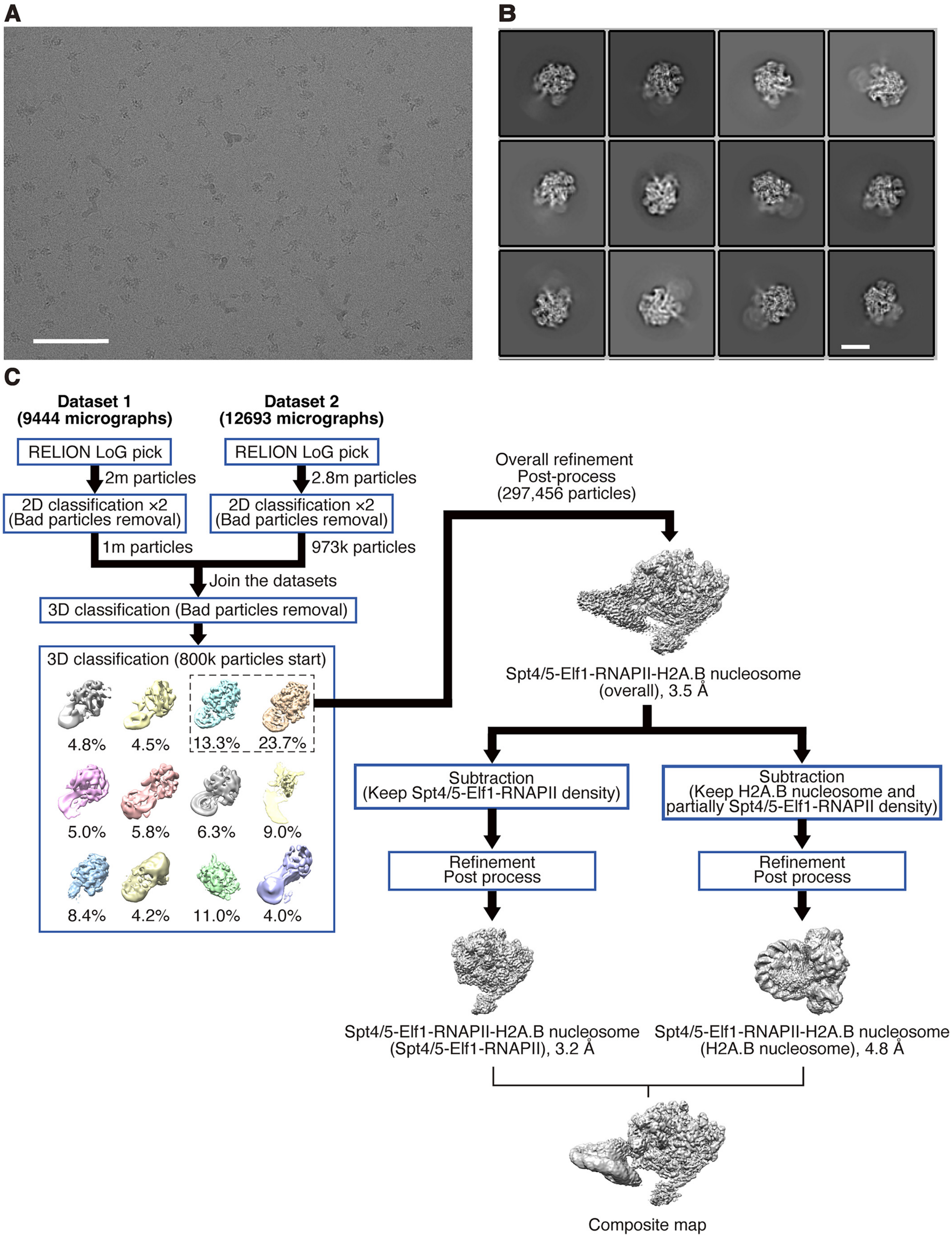
A
B
C
Dataset 1
(9444 micrographs)
Dataset 2
(12693 micrographs)
RELION LoG pick
RELION LoG pick
2m particles
2.8m particles
2D classification ×2
(Bad particles removal)
2D classification ×2
(Bad particles removal)
1m particles
973k particles
Join the datasets
3D classification (Bad particles removal)
3D classification (800k particles start)
4.8%
4.5%
13.3%
23.7%
5.0%
5.8%
6.3%
9.0%
8.4%
4.2%
11.0%
4.0%
Overall refinement
Post-process
(297,456 particles)
Spt4/5-Elf1-RNAPII-H2A.B nucleosome
(overall), 3.5 Å
Subtraction
(Keep Spt4/5-Elf1-RNAPII density)
Subtraction
(Keep H2A.B nucleosome and
partially Spt4/5-Elf1-RNAPII density)
Refinement
Post process
Refinement
Post process
Spt4/5-Elf1-RNAPII-H2A.B nucleosome
(Spt4/5-Elf1-RNAPII), 3.2 Å
Spt4/5-Elf1-RNAPII-H2A.B nucleosome
(H2A.B nucleosome), 4.8 Å
Composite map

 © The Author(s)

Figure EV3. Cryo-EM analysis of the EC-H2A.B nucleosome complex paused at the SHL(−1) position.

(**A**) Representative micrograph of the EC-H2A.B nucleosome complex paused at the SHL(−1) position. Scale bar: 100 nm. (**B**) Representative 2D class averages for the final map of the EC-H2A.B nucleosome complex paused at the SHL(−1) position. Scale bar: 10 nm. (**C**) Workflow of the image processing of the EC-H2A.B nucleosome complex paused at the SHL(−1) position.

© The Author(s)

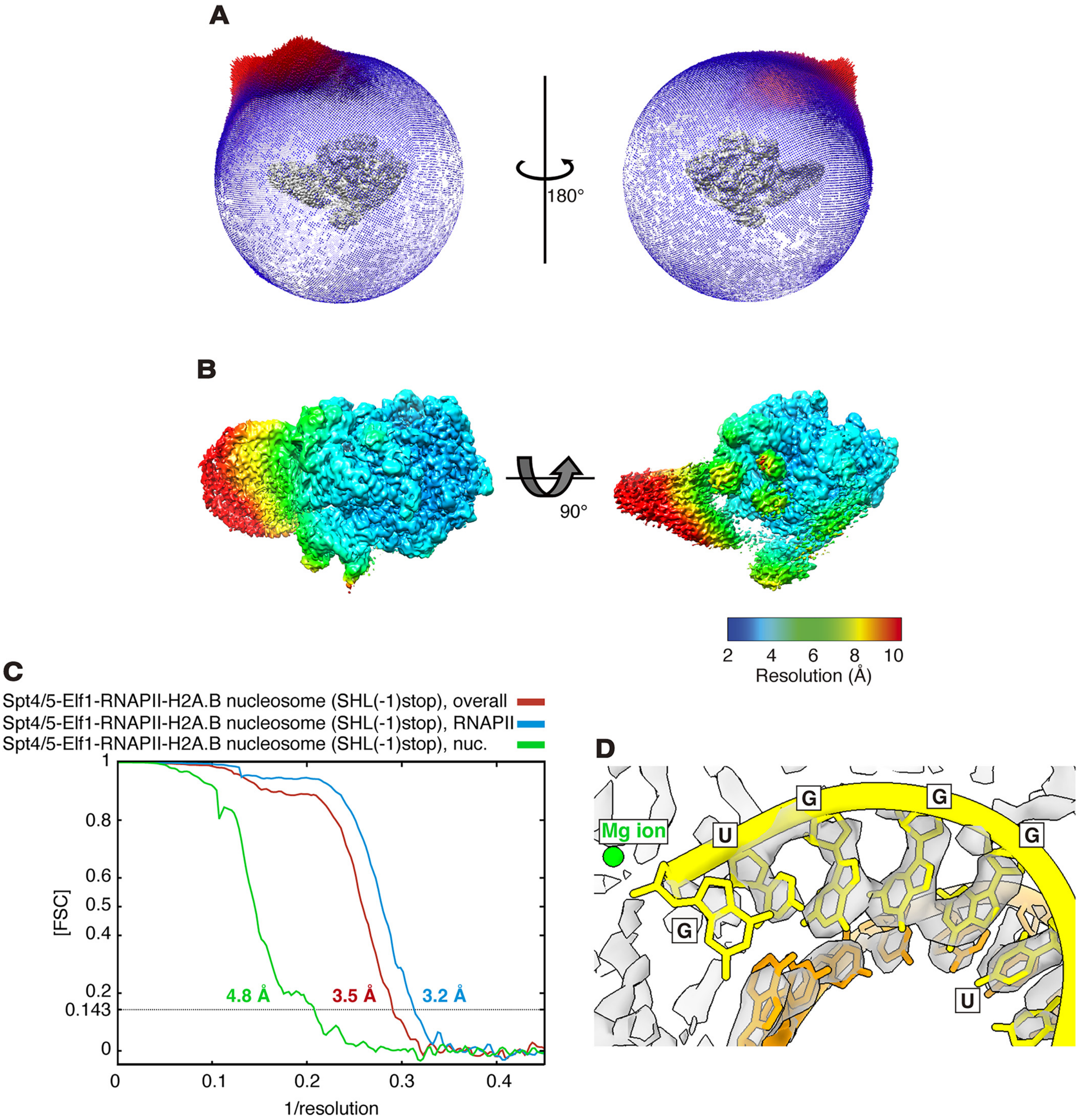

Figure EV4. Cryo-EM map and model qualities of the EC-H2A.B nucleosome complex paused at the SHL(−1) position.

(**A**) Angular distribution plots of the EC-H2A.B nucleosome complex paused at the SHL(−1) position. (**B**) Local resolution map of the EC-H2A.B nucleosome complex paused at the SHL(−1) position. (**C**) Fourier Shell Correlation (FSC) curve of the EC-H2A.B nucleosome complex paused at the SHL(−1) position. The final resolutions of the structures were estimated at 3.5 Å (overall), 3.2 Å (Spt4/5-Elf1-RNAPII), and 4.8 Å (nucleosome) (FSC = 0.143). (**D**) Close-up view of the RNA in the RNAPII catalytic center. Nascent RNA, template DNA, and magnesium atom are colored yellow, orange, and green, respectively. The RNAPII pausing site is determined based on the RNA sequence (5′-UGGGUG-3′) shown in the panel.

 © The Author(s)

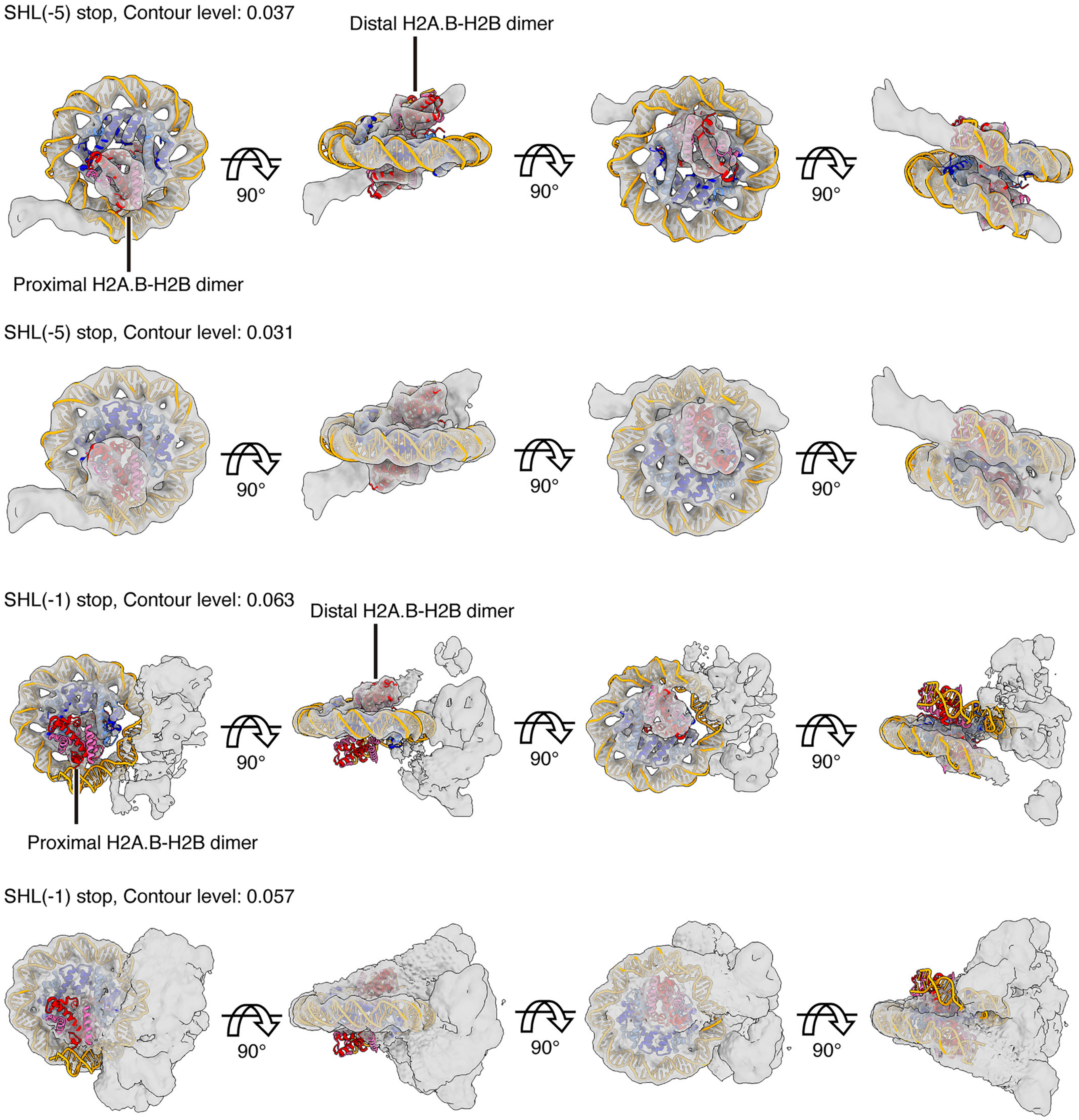

Figure EV5. Cryo-EM maps of the nucleosome regions in the EC-H2A.B nucleosome complexes paused at the SHL(−5) and SHL(−1) positions.

Focused refinement maps of the nucleosome regions with different contour levels. The atomic model of the H2A.B nucleosome (PDB: 6M4G) is superimposed.
